# Development of a Functional Schwann Cell Phenotype from Autologous Porcine Bone Marrow Mononuclear Cells for Nerve Repair

**DOI:** 10.1155/2012/738484

**Published:** 2012-06-24

**Authors:** Michael J. Rutten, Michael Ann Janes, Ivy R. Chang, Cynthia R. Gregory, Kenton W. Gregory

**Affiliations:** ^1^Providence Health and Services, 9555 SW Barnes Rd., Portland, OR 97225, USA; ^2^OHSU Center for Regenerative Medicine, Oregon Health & Science University, 3181 S.W. Sam Jackson Park Road, Portland, OR 97239, USA; ^3^Oregon Biomedical Engineering Institute, 25999 SW Canyon Creek Rd., Wilsonville, OR 97070, USA; ^4^Portland VA Medical Center, 3710 SW U.S. Veterans Hospital Rd., Portland, OR 97239, USA

## Abstract

Adult bone marrow mononuclear cells (BM-MNCs) are a potential resource for making Schwann cells to repair damaged peripheral nerves. However, many methods of producing Schwann-like cells can be laborious with the cells lacking a functional phenotype. The objective of this study was to develop a simple and rapid method using autologous BM-MNCs to produce a phenotypic and functional Schwann-like cell. Adult porcine bone marrow was collected and enriched for BM-MNCs using a SEPAX device, then cells cultured in Neurobasal media, 4 mM L-glutamine and 20% serum. After 6–8 days, the cultures expressed Schwann cell markers, S-100, O4, GFAP, were FluoroMyelin positive, but had low p75(NGF) expression. Addition of neuregulin (1–25 nM) increased p75(NGF) levels at 24–48 hrs. We found ATP dose-dependently increased intracellular calcium [Ca^2+^]*_i_*, with nucleotide potency being UTP = ATP > ADP > AMP > adenosine. Suramin blocked the ATP-induced [Ca^2+^]*_i_* but *α*, *β*,-methylene-ATP had little effect suggesting an ATP purinergic P2Y2 G-protein-coupled receptor is present. Both the Schwann cell markers and ATP-induced [Ca^2+^]*_i_* sensitivity decreased in cells passaged >20 times. Our studies indicate that autologous BM-MNCs can be induced to form a phenotypic and functional Schwann-like cell which could be used for peripheral nerve repair.

## 1. Introduction

There is now sufficient evidence to suggest that the addition of exogenous Schwann cells to injured peripheral nerves can play an important role in the repair of the nerve [1, 2]. The challenge though is the ability to produce sufficient numbers of viable Schwann cells for use in clinical studies. Cell sources used to generate Schwann-like cells have included bone marrow mesenchymal stromal cells (MSCs) [3–7], embryonic stem cells [8], adipose stem cells [9, 10], and umbilical cord MSCs [11]. In some studies, Schwann or nerve cells were induced from MSCs using a chemical and growth factor induction mixture [3–7]. However, the chemical induction of cells with nerve-like morphology from MSCs has been shown to be the result of osmotic cell shrinkage along with changes in the cytoskeleton, and that once the chemical mixture was withdrawn from the cells they immediately reverted back to their original shape [12–15]. Although MSCs may have a supporting immunomodulatory role in nerve repair [16], several studies now question the use of a chemically differentiated MSCs as a source of Schwann cells, which raises the issue that other Schwann cell production protocols are needed [17]. 

It is well known that differentiated Schwann cells express purinergic-G protein receptors that when activated by an agonist such as ATP produce a transient increase intracellular calcium Ca^2+^ ([Ca^2+^]_*i*_). This ATP-induced [Ca^2+^]_*i*_ change has been reported for neonatal and adult Schwann cells [18, 19] as well as for isolated peripheral nerve Schwann cells [20, 21]. The exact role ATP and [Ca^2+^]_*i*_ have in normal Schwann cell function is still being examined, but it is believed that ATP can act as a regulatory signaling molecule between Schwann cells and neurons to control their activity [2, 22]. Therefore, in addition to their morphology, a criterion for characterizing differentiated Schwann cells is their physiological response to ATP-purinergic signaling.

An important objective for this study was to develop a media formulation and a rapid procedure for stimulating the differentiation of porcine BM-MNCs into Schwann-like cells. Currently, the approaches to generate myelin-like cells from other cell types such as bone marrow MSCs require multistep culturing procedures that necessitate several weeks to produce the final cell product [6, 7, 9, 11, 23–26]. The development of a simple and rapid culture method for generating autologous Schwann-like cells would have great therapeutic importance given suggestions that early intervention with treatments for damaged nerves may result in better outcomes [27]. The use of autologous BM-MNCs to generate Schwann-like cells would also be advantageous in that the use of autologous cells eliminates the concerns of cell loss due to immune rejection [28]. Also, as porcine physiology and nerve anatomy are considered to be closer to that of humans than are those of small animals [29, 30], the use of porcine BM-MNCs for Schwann cell derivation will facilitate future translational preclinical studies of peripheral nerve repair.

In the following study, we examined the feasibility of using autologous BM-MNCs to produce cells with morphologic and physiologic characteristics consistent with Schwann cells, which then could be used for peripheral nerve repair.

## 2. Methods

### 2.1. Bone-Marrow Harvest and Purification

All bone marrow samples were collected from 3-4 month male or female domestic Yorkshire swine (Swine Center, Washington State University, Pullman, WA). The procedures of handling and care of the animals were strictly performed in accordance with the 2004 National Research Council “Guide for the Care and Use of Laboratory Animals” and following protocol approval by the Institutional Animal Care and Use Committee (IACUC) of the Legacy Clinical Research and Technology Center, Legacy Health System, Portland, OR. Under local anesthesia, 37 ml of porcine bone marrow was aspirated from each donor's iliac crest into a syringe containing 5 ml of heparin (1000 USP units/ml). The bone marrow was then transferred into a 150 ml transfer bag (Baxter, Deerfield, IL) containing 8 ml of citrate-phosphate dextran (Sigma, St. Louis, MO). The bone marrow transfer bag was then connected through a 40 *μ*m Pall blood transfusion filter (Fisher) to a CS-900 SEPAX cartridge kit (CS-900, Biosafe America, Houston, TX). This cartridge contains a wash-buffer bag that was filled with Hanks' balanced salt solution with cations (HBSS), (Invitrogen), a density gradient solution/waste bag that was filled with 100 ml of Histopaque-1077 (Sigma, St. Louis, MO), and a third 150-mL transfer bag (Baxter, Deerfield, IL) used to recover the purified BM-MNCs. The bone marrow cells were then processed using an automated, completely enclosed cell processing device (SEPAX, Biosafe America) [31]. The final purified bone marrow mononuclear cell product (BM-MNC) was collected in HBSS, and the BM-MNC cell numbers were counted with a Beckman Z2-Coulter Counter (Brea, CA).

### 2.2. Cell Culture and Schwann Cell Differentiation

Two different media protocols were assessed to find an appropriate Schwann-cell differentiation media for the BM-MNCs. The first protocol was a modification of a multistep *β*-mercaptoethanol (*β*-ME), retinoic acid, and growth factor method [7]. For these experiments the BM-MNCs were plated at 2 × 10^5^/cm^2^ in T-25 flasks and cultured in *α*-MEM supplemented with 2 mM L-glutamine, and penicillin/streptomycin solution (Gibco) with 10% fetal bovine serum (FBS; Invitrogen), at 37°C in a 5% CO_2_ incubator for 48 hours. The cells were then switched to an *α*-MEM media containing 1 mM *β*-ME with penicillin/streptomycin and incubated for other 24 hours. Subsequently, the media was replaced with alpha-MEM, 10% FBS, and 35 ng/ml all-trans-retinoic acid (Sigma, St Louis, MO) for the final 3 days of culture. 

 The second media protocol was based on a modification of previously described reports of the success of neurobasal media for establishing neuronal cultures [32, 33]. For these experiments, BM-MNCs were plated at 2 × 10^5^/cm^2^ in T-25 flasks in neurobasal medium (no. 21103-049, Invitrogen) supplemented with 4.0 mM L-glutamine, penicillin/streptomycin, and 20% serum. After 48 hours, the media was removed and replaced with the fresh neurobasal media containing the supplements and changed every other day. Subconfluent cultures were passaged using trypsin/EDTA (Gibco), and when appropriate the cells were replated in T-75 flasks or 35 mm glass bottom dishes (W20, Bioscience Tools). The experiments were done using BM-MNC-derived Schwann cells from passages 1–5, with the addition of using cells from passages 20–25 for those experiments assessing the impact of high passage number on cell characteristics. Human sNF02.2 Schwann cells (CRL-2885, American Type Culture Collection, Manassas, VA) were cultured identically to the BM-MNCs and used as positive controls.

The gross morphological differentiation of the bonemarrow stem cells was monitored by light microscopy and documented by photography using a Leica DM-IRB inverted microscope with a Nikon CoolPix 35 mm camera.

### 2.3. Cell Culture Treatment with Neuregulin-1 (NRG1-III)

After six days in the neurobasal media with supplements, some of the cultures were treated with the NRG1-III, an isoform of Neuregulin-1 (ab23378; Abcam, San Francisco, CA) at doses of 1,10, or 25 nM. After 48 hours of treatment, the cultures were then tested for changes in p75(NGF) receptor expression and increases in FluoroMyelin staining.

### 2.4. Immunofluorescence Staining

For immunofluorescent staining, differentiated BM-MNCs and sNF02.2 Schwann cells were grown on Lab-Tek 8-multiwell glass chamber slides or 35 mm glass bottom dishes (W20, Bioscience Tools). At the appropriate times, the cells were fixed with 2.5% paraformaldehyde (EMS, Hatfield, PA) for 15 min at room temperature, washed 2 X with HBSS, permeabilized with 0.2% Triton X-100 for 1 min, and then blocked for 30 min with Image-iT (Invitrogen). Primary antibodies were added to the cells for an overnight incubation at 4°C. After washing, the secondary antibody was added for 1 hr at room temperature. Following antibody treatment, the cells were stained for 1 min with a 1 : 1000 dilution of DAPI (D1306; Invitrogen) to visualize the nuclei. After DAPI staining, the cells were washed and sealed with the antiquenching agent Cytoseal-60 (Fisher Sci.). Control staining cells were similarly processed without the primary antibody. The primary antibodies included monoclonal anti-S100 beta (Thermo Scientific, Rockford, IL), monoclonal anti-O4 (clone 81, MAB345, Millipore, Temecula, CA), monoclonal anti-Glial Fibrillary Acidic Protein (GFAP, Z0334, DAKO, Carpinteria, CA), monoclonal anti-p75(NGF) receptor (MA1-20167, Affinity Bioreagents), which were at 1 : 250 dilutions. The secondary antibodies used were the green Alexa Fluor-488 goat anti-mouse and red Alexa Fluor-568 goat anti-mouse (A-11029, A-11004, Molecular Probes), which were used at 1:500 dilutions.

Immunofluorescent staining of the cells was photographed using a Zeiss 510 Meta multiphoton confocal microscope equipped with an Axiovert 200M inverted microscope (Zeiss, Thornwood, NY) with 20 X/0.75 NA Plan-Apochromat, 40 X/1.3 NA oil EC Plan Neo-Fluar, or 63 X/1.4 NA oil Plan-Apochromat objectives. Excitation/emission wavelengths at 488/519 and 578/603 nm were used for the Alexa-Fluor green and red primaries, respectively, FluroMyelin with 558/654 nm, and DAPI at 358/461 nm.

### 2.5. Fluorescence Staining for Cell Myelin

Fluorescence staining for myelin was done using FluoroMyelin Red (F34652, Molecular Probes). Differentiated bone marrow cells were fixed with 2.5% paraformaldehyde for 15 min, washed 2 X with HBSS, then a 1 : 300 dilution of the FluroMyelin solution was added for 20 min. The FluroMyelin solution was removed and the cells were washed 2 X with HBSS. They were stained for 1 min with a 1 : 1000 dilution of DAPI and then sealed with ProLong Gold antifade reagent (Invitrogen).

### 2.6. ATP-Induced Intracellular Calcium [Ca^2+^]_*i*_ Measurements

#### 2.6.1. Live-Cell Confocal Imaging

For live-cell confocal imaging of [Ca^2+^]_*i*_ changes, preconfluent, low passage (1–5 passages), BM-MNC-derived Schwann cells were plated on 35 mm glass dishes at 4 × 10^6^ cells/ml in complete neurobasal media. After two days in culture, the media was removed and the cells were loaded with 4 *μ*M of Fluo-4/AM (special packaging F-14201, Molecular Probes) in serum-free media for 30 minutes at 37°C in a CO_2_ incubator. For each experiment, the Fluo-4/AM fluorescent dye was freshly prepared in culture grade DMSO (D2650, Sigma). After Fluo-4 loading, the cultured cells were rinsed in HBSS and incubated for another 30 min in Schwann cell media. The Schwann cell media was then removed and the cultures switched to a mammalian Ringer solution consisting of 137 mM NaCl, 4 mM KCl, 25 mM NaHCO_3_, 2 mM KH_2_PO_4_, 15 mM HEPES, 1 mM MgSO_4_, 2 mM CaCl_2_, 25 mM glucose, pH 7.4. A calcium-magnesium-free Ringer solution was also used where CaCl_2_ and MgSO_4 _ were eliminated and substituted with NaCl along with the addition of 2.0 mM EGTA-EDTA, pH 7.4. The 35 mm glass dishes containing the cells were put into a temperature and CO_2_ controlled mini-chamber system (no. LPPCP1-W, Bioscience Tools, San Diego, CA) that was mounted on the stage of a Zeiss Axiovert 200 M microscope. For pharmacological examination of the presence of P2Y-receptor-mediated intracellular Ca^2+^ responses, ATP agonist and blockers were added at varying concentrations to the Ringer solution with a syringe microinjection system. Confocal images of the cells were acquired at an emission wavelength of 505 nm after excitation at 488 nm in time intervals ranging from 0.5–2.0 seconds. The [Ca^2+^]_*i*_ responses were defined as F/F_0_, where F was the fluorescence at any given time and F_0_ was the initial basal fluorescence and the fluorescence intensity of selected regions of interest plotted against time, % ΔF/F_0_ [34, 35].

#### 2.6.2. Multiwell Plate Assay of [Ca^2+^]_*i*_

For simultaneous comparisons of the effects of ATP agonists and antagonist on cells, BM-MNC-derived Schwann cells were seeded into clear-bottomed, black walled, 96-multiwell plates (#3603, Corning) at a density of 2 × 10^4^ cells/well and cultured for two days in neurobasal media with 4 mM L-glutamine, 20% serum, and antibiotics. The media was removed and replaced with mammalian Ringer solution and 0.1% serum containing Fluo-4/NW and probenicid (to reduce dye efflux) as recommended by the manufacturer instructions (F36205; Molecular Probes). The cells were then incubated at 37°C for 30 m in a CO_2_ incubator, then for another 15 min at room temperature. After this time, the ATP agonists, antagonists, and ionomycin (as a positive control) were added to the appropriate wells of the 96-well plate and then the plate was put into a fluorescence plate reader (Tecan Systems Inc., San Jose, CA). Using excitation/emission filters of 485 nm/535 nm, the Fluo-4 fluorescence was recorded at 15 second intervals over a 5-minute period, then the data downloaded and saved into an Excel spreadsheet.

### 2.7. Schwann Cell Growth Studies

Cell growth of the BM-MNC-derived Schwann cells was done using a modification of the CyQuant-NF (Molecular Probes) fluorescence assay. At the appropriate times, low passage (2–5 passages) and high passage (20–25 passages) of the BM-MNC-derived Schwann cells were trypsinized and seeded into clear-bottomed, black walled, 96-multiwell plates at a density of 1 × 10^4^ cells/well. The cells were then cultured for four days in neurobasal media with 4 mM L-glutamine, 20% serum, and antibiotics with media changes occurring every day. At the appropriate times, the media was removed and 50 *μ*L of a 1 X CyQuant-NF dye binding solution prepared in Hanks buffer with cations was added to each well. The 96-well plate was incubated at 37°C for 45 minutes, and then read on a Tecan fluorescence microplate reader using excitation/emission filters of 485 nm/535 nm. Blank wells without cells containing only the CyQuant-NF dye served as background controls. After reading the plates, the data were then downloaded and saved into an Excel spreadsheet. The blank wells were subtracted from the test samples and the proliferation results graphed and expressed as the change in relative fluorescent units (RFUs) over time. Doubling time was calculated according to previously published methods [36] using the linear portion on the exponential part of the growth cure and the formula DT = ((T2 − T1)*log⁡(2)/log⁡(A2/A1)), where DT: doubling time, T2-T1: time period, and A2 and A1 the absorbance's at T2 and T1, respectively.

### 2.8. Statistical Analysis

Data were analyzed for statistical significance using Sigma Stat software (Systat Software Inc., San Jose, CA). Statistical tests were performed using either the Student *t*-test between pairs or the ANOVA with the Bonferroni *t*-test for multiple pairwise comparisons. All results are presented as mean ± SEM, and the use of “*n*” in our study is equal to the number of individual BM-MNC isolations. 

## 3. Results

### 3.1. Differentiation Media Studies and Schwann Cell Markers

**Figure 1:**
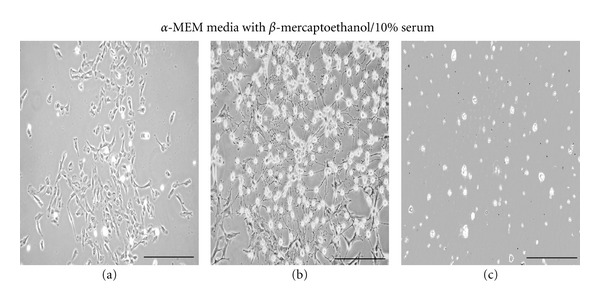
Series of light micrographs showing that porcine BM-MNCs failed to thrive in the chemical induction media. The BM-MNCs were initially plated in *α*-MEM, 2 mM L-glutamine, and 10% FBS media for 48 hours (a). Cultures were then treated with 1 mM *β*-mercaptoethanol added for 24 hours (b); (c) represents cultured cells in *α*-MEM media for four days after being treated for 24 hours in *β*-mercaptoethanol. Scale bar, 250 *μ*m.

In our initial studies, we assessed two different approaches to find an appropriate Schwann-cell differentiation media for the BM-MNCs. In the first case, we used a previously described chemical/growth factor differentiation method [7]. For these experiments, the BM-MNCs were initially cultured in *α*-MEM media with 2 mM L-glutamine and 10% FBS for 48 hours (Figure 1(a)), then switched to *α*-MEM media containing the induction compound 1 mM *β*-mercaptoethanol for 24 hours (Figure 1(b)). However, we found that after 24 hours of culture in medium containing the *β*-mercaptoethanol that most of the cells had either elongated in shape or became rounded and were detaching from the culture plate (Figure 1(b)). We attempted to rescue these *β*-mercaptoethanol-treated cells with fresh *α*-MEM media containing 2 mM glutamine and 10% FBS but found within four days that all the previously—*β*-mercaptoethanol-exposed cells were lysed or nearly dead (Figure 1(c)).

**Figure 2:**
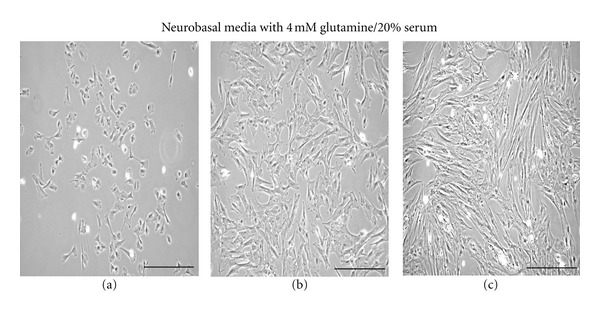
Series of light micrographs showing porcine BM-MNCs that differentiated into elongated cells in neurobasal media. The BM-MNCs were plated in neurobasal media with 4 mM L-glutamine and 20% serum and the cells photographed at 2-days (a), 6-days (b), and 8-days (c). Scale bar, 250 *μ*m.

In the second case, we used neurobasal media as the base component. After a series of preliminary experiments using varying combinations of neurobasal media with L-glutamine and serum, we found that neurobasal media supplemented with 4 mM L-glutamine and 20% serum was optimal for supporting BM-MNC-derived growth into Schwann-like cells (Figure 2). Compared to the chemical differentiation media (Figure 1), the BM-MNC-derived Schwann cells became nearly confluent after 8 days in culture (Figure 2). Of interest was the observation that the cells growing in the neurobasal media began to cluster into parallel-elongated cellular arrays as they neared confluency (Figure 2(c)). 

**Figure 3:**
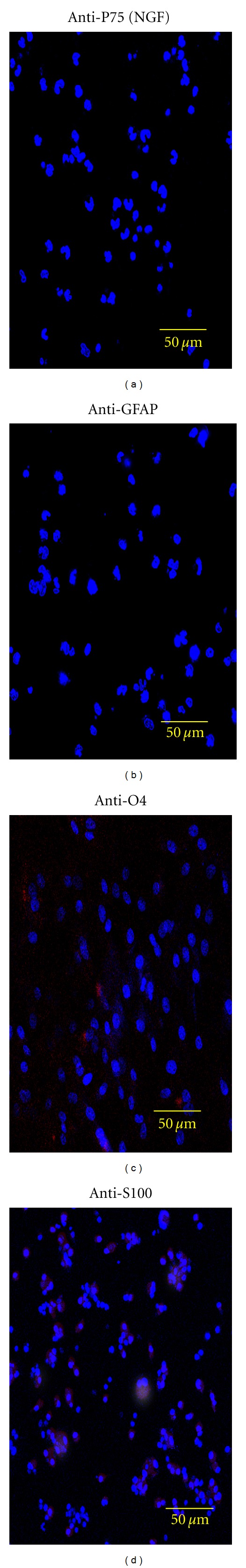
Series of confocal photographs showing that freshly isolated porcine BM-MNCs do not express Schwann cell markers in neurobasal media. The cells were found to be negative staining for the Schwann cell markers p75(NGF), GFAP, O4, and S100 (a, b, c, d). Nuclei (blue) are stained with DAPI.

**Figure 4:**
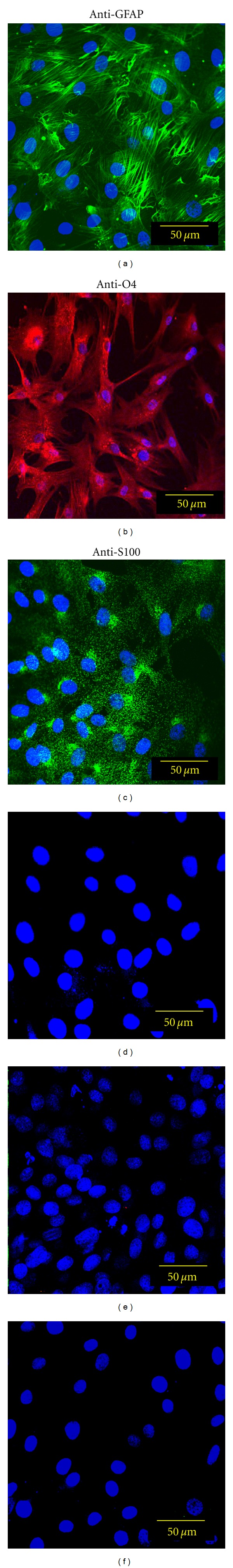
Series of confocal photographs showing that BM-MNCs differentiate in neurobasal media to express Schwann cell markers. There was positive immunofluorescence staining for the Schwann cell markers GFAP, O4, and S100 in 8-day-old BM-MNC-derived Schwann cell cultures (a, b, c); (d, e, f) are control cultures stained with the secondary antibody only. Nuclei (blue) are stained with DAPI.

Immunofluorescence analysis of 24-hour cultured BM-MNCs demonstrated negative to very weakly positive staining for the Schwann cell markers p75(NGF) receptor, GFAP, O4, and S100 (Figure 3). However, after 8 days of culture in the neurobasal differentiation media, the BM-MNC-derived Schwann-like cells were strongly positive for the Schwann cell markers GFAP, O4, and S100 (Figure 4), but remained negative for the Schwann cell p75(NGF) receptor (data not shown).

### 3.2. NRG1 Regulation of p75(NGF) Receptor Expression and Myelin-Lipid Detection

**Figure 5:**
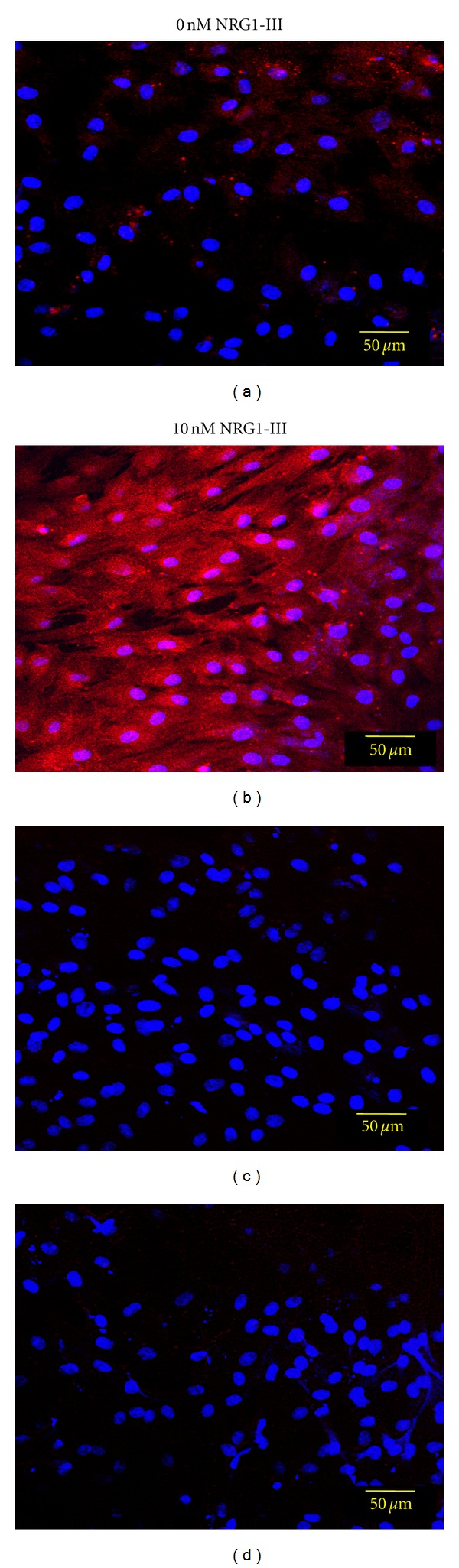
Confocal photographs showing that BM-MNCs cultured in neurobasal media supplemented with NRG1-III express the p75(NGF) receptor. The addition of 10 nM NRG1-III for 48-hrs to 6-day-old cultures increased the expression of p75(NGF) (b) over untreated control cells (a); (c, d) are control cultures stained with only the secondary antibody. The nuclei (blue) were stained with DAPI.

**Figure 6:**
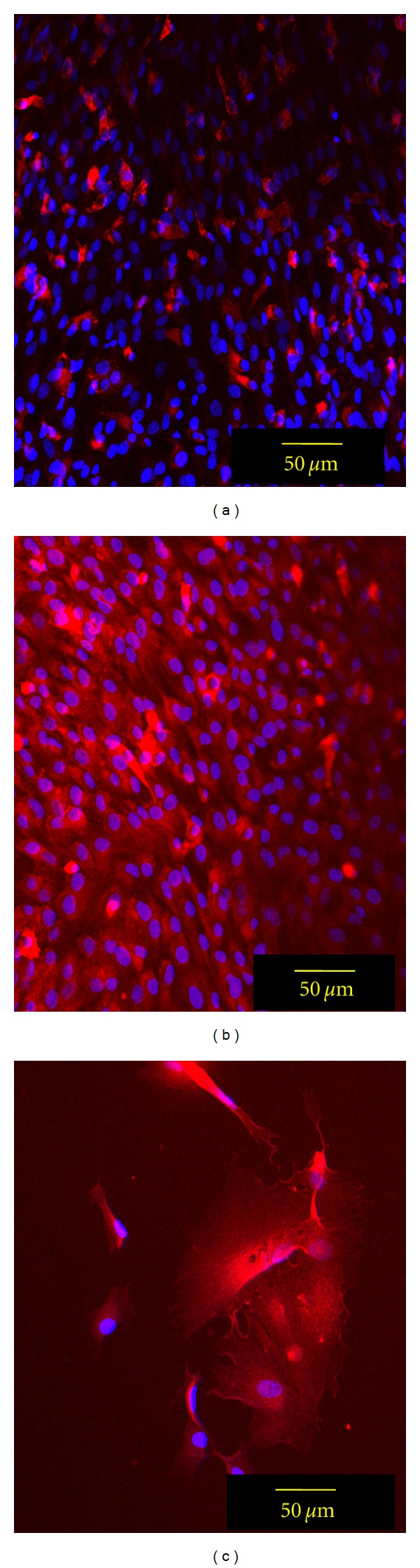
Series of confocal photographs showing BM-MNCs cultured in Neurobasal media supplemented with NRG1-III increase the expression of myelin lipid. Using the myelin-lipid marker, FluoroMyelin, there was an increase in fluorescence staining (red) in the 10 nM NRG1-III 48-hr treated 6-day old cultures of BM-MNC-derived Schwann cells (b) compared to untreated cells (a). A human Schwann cell line was used as a positive control (c); nuclei (blue) are stained with DAPI.

In the previous experiments, we found that the neurobasal media was able to increase the expression of the Schwann cell markers GFAP, O4, and S100, but it was without effect on changing p75(NGF) receptor expression. It is known that neuregulin, especially the NRG1 type-III isoform, is involved in Schwann cell development and differentiation [37, 38], and it is capable of increasing p75(NGF) receptor expression [39] and cholesterol biosynthesis [40]. For the next series of experiments, we supplemented the neurobasal medium with various doses (1 nM, 10 nM, 25 nM) of NRG1-III and cultured the BM-MNCs for 5 days to determine if p75(NGF) levels could be increased. We found that 10 nM NRG1-III was the optimal dose for increasing p75(NGF) receptor levels within BM-MNC-derived Schwann cells (Figure 5). The same 10 nM dose of NRG1-III was also able to increase a cellular myelin lipid product within the BM-MNC-derived Schwann cells over a 2-day period as detected by the FluoroMyelin probe (Figure 6). As a control, a human Schwann cell line known to possess the p75(NGF) receptor [41] was stained with FluoroMyelin and found to have a similar level of fluorescence as the NRG1-III-treated BM-MNC-derived Schwann cells (Figure 6). As a result of these experiments, the addition of NRG1-III to the cultured cells was done for the remainder of this study, including the functional characterization of the [Ca^2+^]_*i*_ responses.

### 3.3. ATP-Induced Intracellular Calcium [Ca^2+^]_*i*_ Measurements

**Figure 7:**
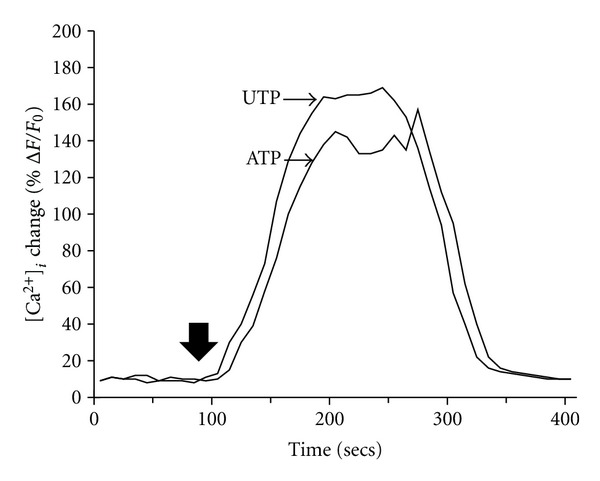
BM-MNC-derived Schwann cells increase [Ca^2+^]_*i*_ in response to UTP or ATP. BM-MNC-derived Schwann cells were loaded with Fluo-4 then either 25 *μ*M UTP or ATP was added (large arrow). Note the near identical nucleotide agonist-induced [Ca^2+^]_*i*_ response to both nucleotides suggesting the presence of a P2Y purinergic receptor.

**Figure 8:**
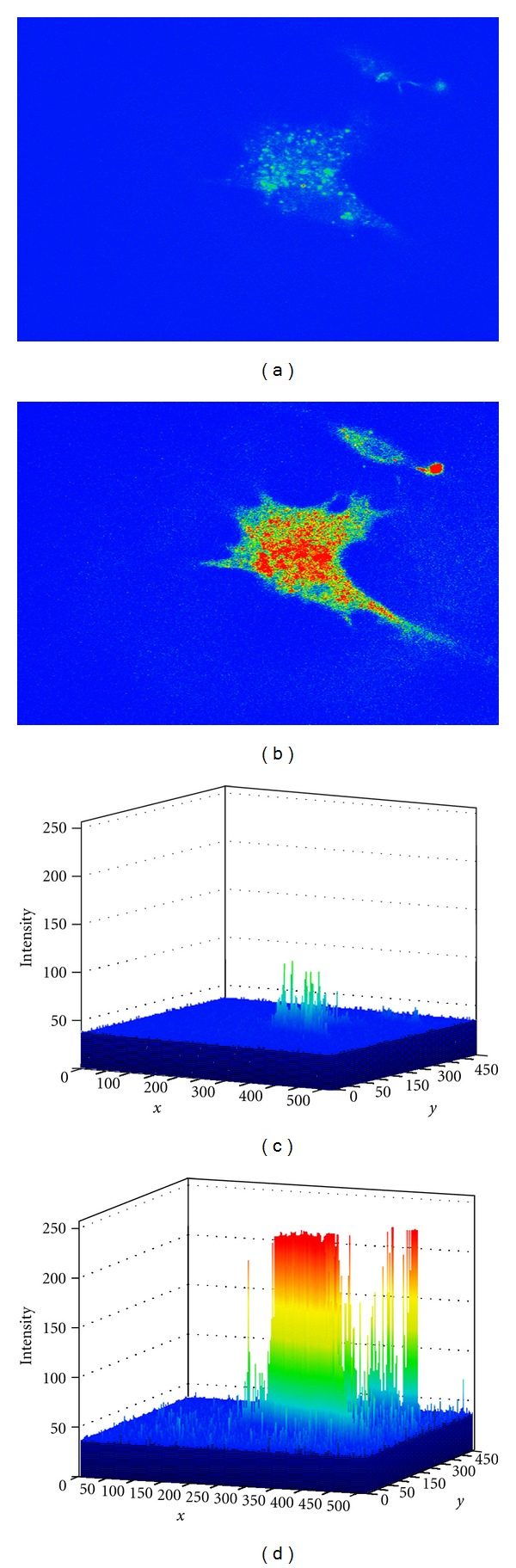
Photographs taken from confocal live-cell imaging of Fluo-4 loaded BM-MNC-derived Schwann cells before (a) and after (b) the addition of 25 *μ*M ATP for 3 minutes. Note the rapid ATP-induced increase in cytoplasmic [Ca^2+^]_*i*_ as indicated by the increase in cellular fluorescence (red) (b); (c, d) represent intensity maps of the relative increase in [Ca^2+^]_*i*_ of the BM-MNC-derived Schwann cell by 25 *μ*M ATP as shown in (a, b), respectively.

**Figure 9:**
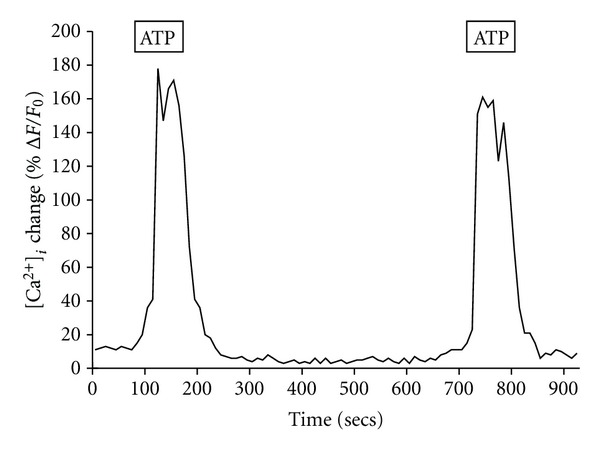
BM-MNC-derived Schwann cells respond to repeated ATP challenges. A representative graph showing the changes in [Ca^2+^]_*i*_ of BM-MNC-derived Schwann cells to repeated exposure of 25 *μ*M ATP. After the first ATP dose and washout, the cells were able to respond again to a second challenge of ATP that produced an [Ca^2+^]_*i*_ transient that was comparable to the first ATP dose.

It is known from a variety of studies that isolated Schwann cells possess a P2Y_2_ purinergic receptor that is sensitive to extracellular ATP, which mobilizes [Ca^2+^]_*i*_ [42, 43]. In our first series of experiments to determine if BM-MNC-derived Schwann cells contained a functional P2Y_2_ receptor, we used confocal imaging with the intracellular calcium-sensitive dye Fluo-4. We found that the addition of either 25 *μ*M UTP or 25 *μ*M ATP to the extracellular medium produced transient increases in [Ca^2+^]_*i*_ of 159 ± 6% F/Fo, and 151 ± 5% F/Fo (*n* = 6), respectively (Figure 7). This ATP-induced intracellular [Ca^2+^]_*i*_ change in the Schwann-like cells can be further visualized as a relative intensity response as shown in Figure 8. Repeated stimulation of the cells with 25 *μ*M ATP resulted in nearly identical peak [Ca^2+^]_*i*_ changes between the first dose (152 ± 7% F/Fo; *n* = 5) and the second dose (145 ± 6% F/Fo; *n* = 5). We found that the repetitive changes in ATP-induced [Ca^2+^]_*i*_ were only observed only if there was a sufficient wash-out time (~5 min) between the applications (Figure 9).

**Figure 10:**
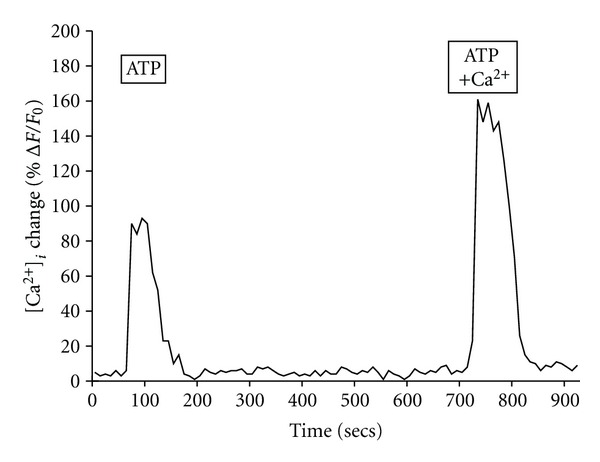
The BM-MNC-derived Schwann cell ATP-induced [Ca^2+^]_*i*_ response is primarily the result of intracellular Ca^2+^ stores. Representative graph shows tracing of the changes in [Ca^2+^]_*i*_ within BM-MNC-derived Schwann cells in response to 25 *μ*M ATP in Ringer without or with calcium.

The fact that the BM-MNC-derived Schwann cells produced nearly similar increases [Ca^2+^]_*i*_ with UTP or ATP suggested the existence of some type of purinergic receptor linked to mobilizing intracellular calcium stores. To examine the contribution of extracellular versus intracellular Ca^2+^ to the total [Ca^2+^]_*i*_ response, we performed a series of experiments using Ca^2+^-free Ringer with 2.0 mM EGTA-EDTA. Using Ca^2+^-free Ringer solution, the BM-MNC-derived Schwann cell ATP-induced [Ca^2+^]_*i*_ response was reduced to 97 ± 7% F/Fo (*n* = 5) as compared to 157 ± 5% F/Fo (*n* = 5) using Ringer solution with Ca^2+^. Although the ATP-induced [Ca^2+^]_*i*_ change in Ca^2+^-free Ringer solution was reduced by ~40% (Figure 10), the experiments indicate that the majority of the total ATP-induced [Ca^2+^]_*i*_ response in the BM-MNC-derived Schwann cells was due to intracellular calcium mobilization.

### 3.4. Nucleotide Characterization of [Ca^2+^]_*i*_ Changes in BM-MNC-Derived Schwann Cells

**Figure 11:**
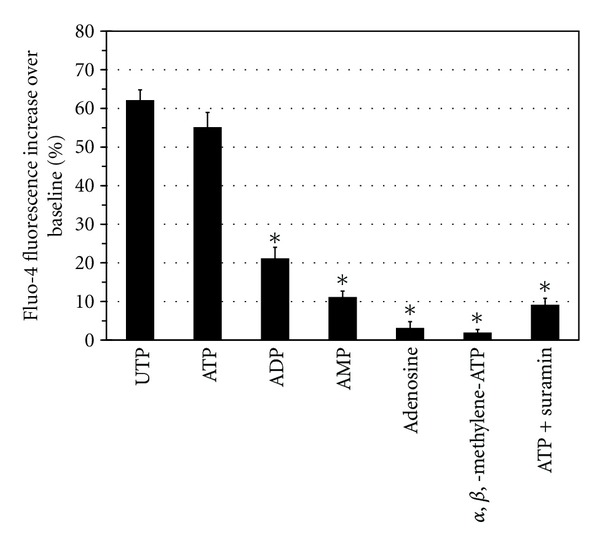
The relative potency of nucleotide agonists for increasing [Ca^2+^]_*i*_ within Fluo-4 loaded BM-MNC-derived Schwann cells is consistent with the presence of a P2Y_2_ receptor. The figure shows the relative potency of purinergic agonists and the nonselective P2 antagonist suramin. The asterisk (∗) represents significantly different (*P* < 0.05) from UTP or ATP values (*n* = 5).

 The BM-MNC-derived Schwann cell responses to UTP and ATP suggested the presence of a putative P2Y purinergic receptor. To determine the exact P2Y receptor subtype, additional experiments were performed using a variety of purine agonists and antagonists. These included 25 *μ*M of the agonists UTP, ATP, ADP, AMP, adenosine, *α*,*β*,-methylene-ATP, or 100 *μ*M of the nonselective P2 antagonist suramin. To allow a pharmacological comparison of all compounds, the Fluo-4/NW assay was performed on the BM-MNC-derived Schwann cells cultured in 96-multiwell plates. We found that the change in [Ca^2+^]_*i*_ was maximal in response to UTP and ATP, and minimal in response to ADP, AMP, and adenosine (*n* = 5; Figure 11). The P2X_1,3,4_ agonist, *α*,*β*,-methylene-ATP also had little to no effect on [Ca^2+^]_*i*_, and the nonselective P2 antagonist, suramin, was able to reduce the ATP-induced [Ca^2+^]_*i*_ (*n* = 5; Figure 11). Overall, this nucleotide agonist and antagonist profile for intracellular Ca^2+^ mobilization indicates the presence of a functional P2Y_2_ purinergic receptor on BM-MNC-derived Schwann cells.

### 3.5. Effects of Cell Passage on Growth, Phenotype, and [Ca^2+^]_*i*_ Changes of BM-MNC-Derived Schwann Cells 

**Figure 12:**
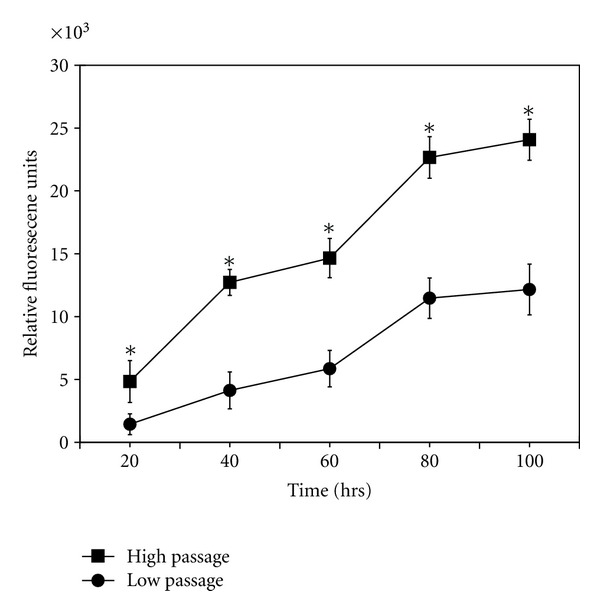
The growth rate of BM-MNC derived Schwann cells is greater in the higher versus lower passage number of cells. The graph shows the growth rates of low 1–5 passages compared to high 20–25 passages of BM-MNC-derived Schwann cells. Cell growth was determined by the CyQuant-NF proliferation assay. The asterisks (∗) represent significantly different (*P* < 0.05) from the low passage cells (*n* = 5).

**Figure 13:**
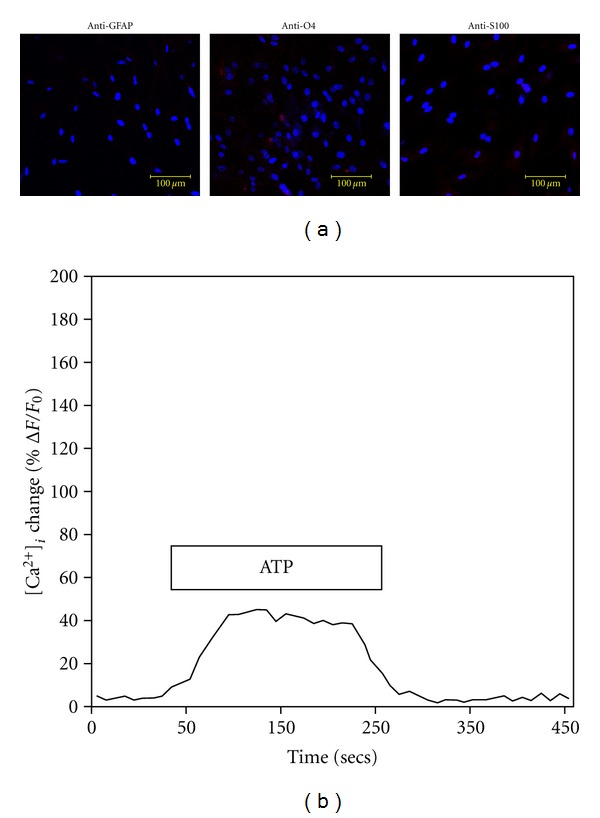
The immunostaining for Schwann cell markers and ATP-responsiveness is decreased in high passage BM-MNC-derived Schwann cells. A representative series of confocal photographs showing Schwann cell marker immunostaining (a) and ATP-responsiveness to [Ca^2+^]_*i*_ (b) within high passage (passage no. 22) BM-MNC-derived Schwann cells. Note there was a reduction in both Schwann cell marker immunostaining (a) and an attenuated ATP-induced [Ca^2+^]_*i*_ response (b).

As a potential therapeutic tool for treating peripheral nerve damage, the BM-MNC-derived Schwann cells would likely have to be expanded in culture before use. However, it is still unclear from the literature how many expansions Schwann cells can undergo before becoming ineffective or potentially tumorigenic [44]. To test the effect of high passage numbers on growth, phenotype, and functionality of BM-MNC-derived Schwann cells, cultures were expanded several times until they reached from 20-25 passages. We found that the high passage BM-MNC-derived Schwann cells had significantly (*P* < 0.05) higher growth rates compared to low passage cells (Figure 12). The average doubling time of the high passage cells was 2.48 ± 0.19 days, while that of the low passage cells was 4.20 ± 0.22 days (*n* = 5). In addition, the high passage cells had little to no immunostaining for the Schwann cell markers GFAP, O4, S100 (Figure 13(a)), and they also had an attenuated ATP-induced [Ca^2+^]_*i*_ response of only 46 ± 6% F/Fo (Figure 13; *n* = 5).

## 4. Discussion

In the current study, we report a method where autologous BM-MNCs were used to generate a phenotypic and functional Schwann-like cell. Unlike other studies [45, 46], these BM-MNC-derived Schwann cells did not need a neuronal feeder line to differentiate, and they could be rapidly expanded after the first passage in culture. Also, compared to the potential for fibroblast contamination of primary cultures of Schwann cells from enzymatically digested tissue [47], fibroblast contamination is less likely to occur in BM-MNC-derived Schwann cells.

An important initial objective for this study was to develop a media formulation for stimulating differentiation of porcine BM-MNCs into Schwann-like cells in a one-step procedure. In the past, several approaches have been used to generate myelin-like cells from bone marrow MSCs based upon multistep culturing methods [6, 7, 9, 11, 23–26]. Typically, these multistep procedures are time consuming in that they can involve culturing the bone marrow cells for 2 days in an *α*-DMEM media, subculturing the cells four times, 1 day of culture in medium containing *β*-mercaptoethanol, 3 days of culture in medium containing all-trans-retinoic acid, and finally 7days of culture in *α*-DMEM containing growth factors [7]. Because it has been suggested that the therapeutic window for treating injured nerves should possibly be soon after injury [27], one goal of our study was to find a simple culture method that could generate autologous Schwann-like cells within a short-time period for use in peripheral nerve repair.

Our initial attempts in using a modification of the *β*-mercaptoethanol, retinoic acid, and growth factor technique were not successful. We found that the porcine BM-MNCs after 2 days of culture in *α*-MEM supplemented with 2 mM L-glutamine and 10% FBS media when treated with 1 mM *β*-mercaptoethanol (*β*-ME) produced a significant morphological elongation of cells that eventually lead to cell lysis with no viable cells observable after eight days of culture. Our findings are in contrast to what others have reported for *β*-mercaptoethanol (*β*-ME) with subcultured rodent or human MSCs, where *β*-ME had relatively no reported morphological or cytotoxic effects [6, 7, 9, 11, 23–26]. However, the contrasting results of our study may be due to procedural differences, in that we treated BM-MNCs with *β*-ME within two days their isolation whereas others treated MSCs with *β*-ME after 4 weeks of subculturing [6, 7, 9, 11, 23–26]. However, our findings of the *β*-ME-induced cytotoxic and elongation effects on our cultured cells do agree with what others have found for other cell types using chemical induction methods [12–15]. That is, *β*-ME treatment of a variety of cells, including primary fibroblasts, keratinocytes, HEK293 cells, PC-12, and subcultured rat MSCs, produces an elongated neuronal-like morphology due to cellular osmotic shrinkage which can be easily reversed once the *β*-ME is removed [12].

Although the early studies by Brewer and colleagues [32] found that neurobasal media with B27 supplements was able to support the growth of neuronal cultures, they also found that neurobasal media containing serum and 3 mM glutamine was more advantageous for growing myelinating glial cells than neurons [32]. In our study, we found that neurobasal media supplemented with 4 mM glutamine and 20% FBS was consistently able to produce within a 7-day period a cell type positive for the Schwann cell markers GFAP, O4, and S-100. The exact reasons why our neurobasal media combination was able to push the BM-MNCs towards this lineage are not known. However, the neurobasal media is somewhat different than other media in that it is modified Dulbecco's/Ham's F12 media in which the osmolality and the concentration of several amino acids have been reduced and ferrous sulfate eliminated [48]. The neurobasal media in combination with high serum, and glutamine may contain several growth and differentiation factors known to be involved in the cell metabolism linked to Schwann cell differentiation [49]. 

Despite our initial success with this neurobasal media formulation, we found little to no immunostaining for the Schwann cell marker p75(NGF) as well as low Fluromyelin staining. Of interest to our studies were the reports showing that the axon-associated neuregulin isoform, specifically NRG1-III, is likely a necessary component for a fully differentiated Schwann cell phenotype [37, 39, 50, 51], as well as playing a key role in myelination synthesis [52–54]. We found that adding soluble NRG1-III to our cultures for several days resulted in specific increases in p75(NGF) receptor expression as well as increased fluorescence for cellular myelin product as detected by FluroMyelin staining. How the soluble NRG1-III RG is acting on the BM-MNC-derived Schwann cells to bring about these changes is unknown. However, the mechanism is likely to be different than those in studies demonstrating that a coculture with neurons is needed to change Schwann cell precursors to a differentiated cell type [46, 52, 55]. The differentiation of a cell precursor to a mature Schwann cell phenotype is a complex process [50, 56], and there likely is crosstalk between the differentiation signaling pathways of NRG1-III/ErbB and the neurotrophins/p75(NGF) complexes [57]. 

 Successful peripheral nerve repair depends upon the delivery of cells not only with an appropriate Schwann-cell-marker phenotype, but with a correct functional phenotype [27, 58, 59]. It is known that adult Schwann and glial cells possess an ATP-sensitive P2Y receptor that is linked to an IP_3_-mediated intracellular Ca^2+^ response [18, 19]. The role of ATP purinergic signaling is important to Schwann and glial cell health since it has been shown to regulate differentiation, proliferation, myelination, and survival of these cells [60]. For our own studies, we were interested in analyzing whether the BM-MNC-derived Schwann cells, which expressed the Schwann cell markers GFAP, O4, S100, and p75(NGF), were also capable of a functional purinergic ATP intracellular Ca^2+^ response. Using confocal imaging and fluorescence microplate reader analysis, we found that the addition of ATP to the cultures was consistently able to produce an increase in [Ca^2+^]_*i*_. This ATP-induced [Ca^2+^]_*i*_ response was primarily generated from intracellular Ca^2+^ stores as removal of extracellular Ca^2+^ from the media only resulted in an ~40% decrease in the total ATP-induced [Ca^2+^]_*i*_ signal. Upon further characterization of the purinergic response in the BM-MNC-derived Schwann cells, we found that the agonist potency was UTP = ATP > ADP > AMP > adenosine. This hierarchy in agonist potency is similar to that reported for other purinergic-induced Ca^2+^ responses in adult Schwann cells [19, 21, 61–64]. The same nucleotide sequence for increasing [Ca^2+^]_*i*_ in our cells has also been shown to be associated with P2Y_2_ receptors in myelinating Schwann cells on isolated intact nerves [65]. We also found that other agonists such as *α*,*β*,-methylene-ATP had little effect on [Ca^2+^]_*i*_ while the P2Y_2_-receptor-blocker suramin attenuated the ATP-induced [Ca^2+^]_*i*_. As summarized by others [43, 60], these particular responses further suggest the presence of a purinergic P2Y_2_ receptor in our BM-MNC-derived Schwann cells. Although the specific biological functions of P2Y receptors in health and disease are still being determined, it has been suggested that P2Y_2_ receptors could be involved in the control, maintenance, and repair of neuromuscular synapses [22]. Overall, it appears that the ATP-activated P2Y_2_-induced [Ca^2+^]_*i*_ change is a highly conserved signaling function since this response can be found in Schwann cells as far down the evolutionary tree as in elasmobranch fish [66].

It is worth noting that the phenotypic marker expression and ATP-induced [Ca^2+^]_*i*_ responses in the cultured BM-MNC-derived Schwann cells were found to decrease at higher cell-culture passage numbers. Particularly, we found immunostaining for the Schwann cell markers GFAP, O4, S100, and p75(NGF) and the ATP-induced intracellular Ca^2+^ response began to decrease after five cell passages, and both parameters were significantly decreased to very low levels once the cells were passaged more than twenty times in culture. We also found that the higher-passage cultures (>20 passages) had faster proliferation rates compared to low-passage cultures (<6 passages). Although cell culture offers the capability of generating large numbers of Schwann cells for regenerative medicine studies, others have cautioned that long-term culture and expansion of the cells cannot only alter their differentiation and functional potential but possibly change these cells into a tumorigenic phenotype [67]. Some studies have used MSCs at different passage numbers for differentiation into Schwann-like cells [3–7]. In general, the ability to consistently subculture MSCs can be variable, and as yet there are few standardized protocols that have been agreed upon for MSC expansion and passaging [14, 15, 68]. For example, it has been shown that porcine MSCs remained pluripotent at <5 passages while MSCs at >15 passages displayed features of cell aging such as loss of differentiation capacity, actin accumulation, reduced substrate adherence, and increases in the senescent lysosomal marker beta-galactosidase activity [69]. For human bone-marrow-derived MSCs, there are also clear differences in the differentiation potential of early fourth-passage versus late ninth-passage MSCs even though the antigenic expression, of the fourth and ninth passage MSCs were similar [70]. In general, it has been suggested that Schwann cells used at >11 passages should not be employed for tissue engineering experiments [71]. The results of our studies would support this recommendation and suggest that lower passage numbers (<6 passages) of BM-MNC-derived Schwann cells should be used for experimental purposes.

In the future, it will be important to test the *in vivo* function of porcine BM-MNC-derived Schwann cells in animal models of peripheral nerve repair. Although many studies of peripheral nerve repair have been performed in small animals such as rodents, swine are considered to provide a more relevant translational animal model since their physiology and nerve anatomy are closer to that of humans [29, 30]. Studies of advanced stem cell transplantation for spinal cord repair also are performed in large animal models, specifically swine and canine models, as again these animals display a closer homology to humans than do rodents and are thought to provide results with greater clinical applicability [72]. As porcine biomaterials are available for nerve repair strategies, porcine BM-MNC-derived Schwann cells can be used in compliment in porcine peripheral nerve repair models. That is, the porcine small-intestinal-submucosa (SIS), which has been successfully used as a scaffold and conduit for nerve repair studies in rodents [73], could easily be seeded with the autologous porcine BM-MNC-derived Schwann cells for use in short- and possibly long-gap nerve repair experiments in swine. Another approach for peripheral nerve repair could be to seed BM-MNC-derived Schwann cells onto a gel-form derived from a powder of porcine decellularized neural matrix [74]. This matrix has the advantage in that it retains neural-specific molecules such as myelin and laminin that are needed for the support of Schwann and nerve cell growth [74, 75]. Thus, the availability of porcine BM-MNC-derived Schwann cells should facilitate clinically relevant studies of peripheral nerve and spinal cord repair.

 In summary, we report a method where phenotypic and functional Schwann-like cells can be produced from autologous BM-MNCs in the absence of a neuronal coculture. Our methods consist of a simple one-step media procedure used throughout the entire culture period, with the cells maintaining phenotypic and functional characteristics of Schwann-like cells through five passages. Finally, because our method allows Schwann-like cells to be derived from BM-MNCs, patients could be treated with Schwann-like cells cultured from their own BM-MNCs thereby eliminating the need for any immunosuppressive therapy as part of the treatment for repairing peripheral nerve defects.
